# Discovering the hidden messages within cell trajectories using a deep learning approach for in vitro evaluation of cancer drug treatments

**DOI:** 10.1038/s41598-020-64246-3

**Published:** 2020-05-06

**Authors:** A. Mencattini, D. Di Giuseppe, M. C. Comes, P. Casti, F. Corsi, F. R. Bertani, L. Ghibelli, L. Businaro, C. Di Natale, M. C. Parrini, E. Martinelli

**Affiliations:** 1 Department of Electronic Engineering, University of Rome Tor Vergata, Rome, Italy; 2 Department of Chemical Science and Technologies, University of Rome Tor Vergata, Rome, Italy; 3 Institute for Photonics and Nanotechnology, Italian National Research Council, 00156 Rome, Italy; 4 Department of Biology, University of Rome Tor Vergata, Rome, Italy; 5 Institute Curie, Centre de Recherche, Paris Sciences et Lettres Research University, 75005 Paris, France

**Keywords:** Image processing, Biomedical engineering

## Abstract

We describe a novel method to achieve a universal, massive, and fully automated analysis of cell motility behaviours, starting from time-lapse microscopy images. The approach was inspired by the recent successes in application of machine learning for style recognition in paintings and artistic style transfer. The originality of the method relies i) on the generation of atlas from the collection of single-cell trajectories in order to visually encode the multiple descriptors of cell motility, and ii) on the application of pre-trained Deep Learning Convolutional Neural Network architecture in order to extract relevant features to be used for classification tasks from this visual atlas. Validation tests were conducted on two different cell motility scenarios: 1) a 3D biomimetic gels of immune cells, co-cultured with breast cancer cells in organ-on-chip devices, upon treatment with an immunotherapy drug; 2) Petri dishes of clustered prostate cancer cells, upon treatment with a chemotherapy drug. For each scenario, single-cell trajectories are very accurately classified according to the presence or not of the drugs. This original approach demonstrates the existence of universal features in cell motility (a so called “motility style”) which are identified by the DL approach in the rationale of discovering the unknown message in cell trajectories.

## Introduction

Cell motility is fundamental for life, along the entire evolutionary tree, being involved in bacteria collective motion^1^, in the morphogenesis of pluricellular organisms^2^, in adult physiological process (such as tissue repair and immune cell trafficking)^3^ and in some pathologies (such as cancer metastasis)^4–7^. Nature evolved a variety of cell motility modes, single-cell or collective, mesenchymal or amoeboid, random or directed, etc. Yet, since the driving force of cell motility is always the active reorganization of the cellular cytoskeleton, it is reasonable to assume that some universal principles of cell motility behaviours have been conserved. We applied machine learning approach to explore this hypothesis exploiting Deep Learning (DL) architecture, by presenting a novel tool called *Deep Tracking*. DL is a recent machine learning framework^8^ developed on the basis of the human brain machine. DL technique learns how to extract the “style” of an atlas of digital images (like the style from an atlas of an artist’s paintings^9,10^) in order to represent a given set of pictures in terms of most relevant quantitative descriptors (i.e., features)^8^. We addressed the question of whether DL could be proficient in extracting the motility styles, i.e. the paintings drawn by cells while moving.

**Figure 1:**
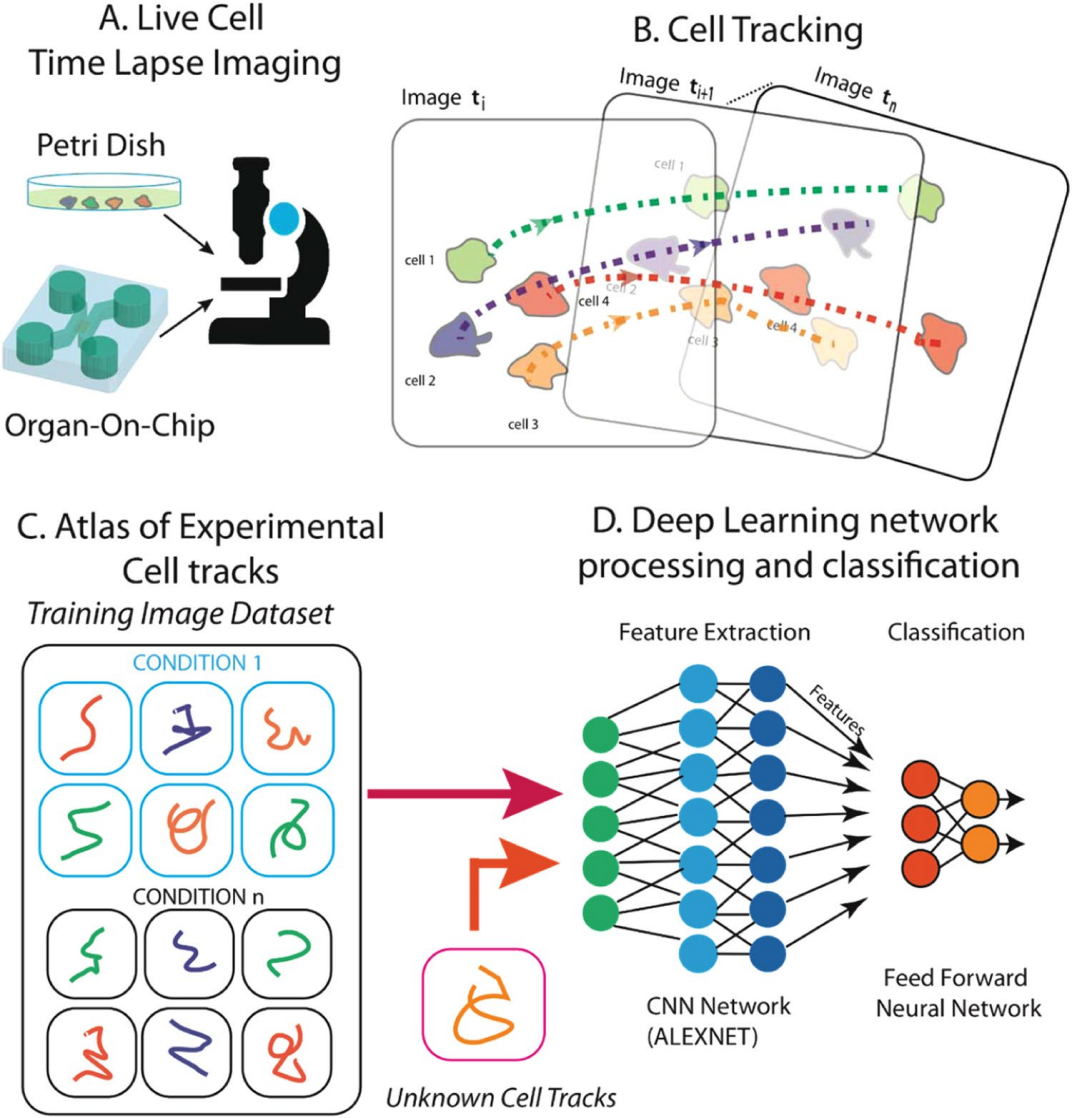
A schematic representation of the proposed method. (**A**) Time-lapse microscopy is used to acquire the video sequence of cells moving in a Petri dish or in an OOC platform. (**B**) Cells are localized and tracked through the video sequence. (**C**) For each cell (or cluster of cells) of interest, an atlas of trajectories is collected for the different biological conditions under consideration. (**D**) Through a pre-trained Deep Learning architecture, i.e., AlexNET, the tool provides a feature representation of each ATLAS in order to perform experiment classification through a predefined taxonomy (e.g., drug vs no-drug).

Typically, cell motility experiments use time-lapse microscopy imaging (Fig. 1). Starting from the image stacks (Fig. 1A), video processing methods are used to track cell trajectories (see the description of the Cell Hunter tool^11,12^ in Steps 2 and 3, Methods section) (Fig. 1B). The first step of our Deep Tracking method relies on the assembly of the individual cell tracks collected for each video (in the range of hundreds) into single images (an atlas), which visually encode a variety of cell motility properties (Fig. 1C). Looking at these motility atlases, like when watching a painting, humans can easily perceive and measure some features, such as the directionality or speed of cell movements; we reasoned that machine learning^13^ will be able to enormously expand the number of features within the atlases to be used for the recognition of motility styles. The second step of Deep Tracking method exploits DL architecture, by using the widely used pre-trained Convolutional Neural Network, AlexNET^14^ (see Steps 4 and 5 In Methods section), to extract these “*unperceivable-to-humans*” features from the visual atlases of each experiment and to use them to classify the cell motility behaviours (Fig. 1D), by implementing the so-called *transfer learning*^15^. For validation of the method we choose a problem particularly relevant in cancer research: the pre-clinical *in-vitro* evaluation of anti-cancer drugs efficacy. We applied Deep Tracking to analyse two motility experiments of cancer cells in presence or not of particular drugs. Further details will be found in Results section.

In the first experiment, breast cancer cells (BT474 cell line, representative of HER2 + breast cancer subtype) were co-cultured in 3D biomimetic gels, within microfluidic devices, with immune cells (PBMCs, peripheral blood mononuclear cells from healthy donors); the addition of an immunotherapy drug, the Trastuzumab (brand name Herceptin), increases cancer-immune cell interactions^12^.

In the second experiment, prostate cancer cells (PC-3 cell line) were treated or not with a standard chemotherapeutic agent (etoposide); these cells naturally form clusters in 2D Petri dishes and the collective motility behaviours within the clusters are inhibited by the drug^16^.

For both experiments, Deep Tracking was able to classify the cells (n > 2·10^3^) in the two categories of treated or untreated with an excellent accuracy (91.5% on average), demonstrating that the deep learning approach is very proficient in recognizing the impact of drug treatments on cell motility behaviours.

The performance of the method, despite the difference of the two scenarios in terms of microenvironment and of the biological diversity of cells involved, supports the notion of the existence of universal features in cell motility, encoded in the visual appearance of cell trajectories, which are identified and exploited by Deep Tracking approach. The possible applications of this innovative and robust method are countless; basically, any biomedical problem involving cell motility and video-microscopy could enormously benefit from the here described Deep Tracking approach, which builds a new and solid bridge between cell biology and artificial intelligence.

The proposed approach is framed in an ever more increasing plethora of work in the field of DL architectures and their applications^15^. DL have demonstrated a clear superiority with respect to existing machine learning approaches across several biomedical and biological fields^17–19^ producing comparable or higher accuracy than previous optimized methods. As one of the most recent example, method in^17^ proposes an architecture for improving cell motility discrimination by combining Deep Convolutional and Recurrence Neural Networks. With respect to^17^, Deep Tracking methodology proposes here many differences with the aim to provide a more effective biology-friendly platform.

First, in the present approach, a cell trajectory is depicted as a 2D image, more adapt for a human-expert visual inspection and understanding than a multichannel time-series approach^20^. As an immediate consequence, the image-coding used in Deep Tracking approach not only allows to perform 2D convolution (in place of 1D convolution independently applied over each coordinate series), but takes advantage of the possibility to apply pre-trained DL architecture (e.g., AlexNET model), exploiting the strength of pre-training on huge quantities of images and categories. Such a possibility opens the frontiers to a more robust use of transfer learning techniques. Transfer learning has proved that large-scale natural image sets^15^ are needed for pre-training models that serve as generic feature extractors for various types of biological images. In this regard, deep learning models have been recently used to predict protein subcellular localization for proteins not originally present in a training set^21^.

In addition, the possibility to use pre-trained net presents many advantages such as that of reducing time-consuming retraining of a new specific net on the new data (or of a part of it), at the risk of overlearning due to a small amount of biological data available. The possibility to use a pre-trained DL allows a high generalizability of the results and support its portability to different biological environment as long as the transferability of features between various closely related and distant learning tasks are investigated and demonstrated.

To overcome, the risk of proposing tailored solutions developed for specific biological tasks, Deep Tracking has been modularly designed allowing the user to embed any tracking tool available (see for example *u track* or *M track* plugin in ImageJ tool^22^) in order to extract trajectories that can be used to generate the atlas for transfer learning. Transfer learning is then used to generate a huge amount of descriptors able to be used in a standard pattern recognition approach (support vector machine) overtaking the question to recover a subset of descriptors in relation to a limited cell behaviour aspect, especially in the rationale of investigating the “unknown message in cell trajectories”.

Another important novelty of Deep Tracking approach relies on avoiding the use of labelled data, a question largely stressed in the review paper^15^. When DL is used to predict trajectories or to segment cells^18^, then manually-collected information are required (manually drawn trajectories or segmented cell shapes). Even if in the literature many attempts emerge to solve this drawback by the use of simulated video data of moving cells^17^, the question remains still debated. In Deep Tracking approach, only the labels of the biological conditions are needed for a subset of experiments (i.e., treated vs untreated), while cell-tracking, image-coding, and transfer learning are entirely performed using an unsupervised method. This means that the algorithms parameters are not tuned according to the label provided by the biologists (treated vs untreated). The only part of the platform that needs the label knowledge is of course the classification task. This approach drastically reduces the effort of experts in favour of an increased usability and usefulness of the platform. Not less relevant, the availability of a large collection of training data needed for DL to learn a novel model is usually hampered by challenges such as local bias, wider standards and legal issues (see discussion in^15^).

As a further new point of view, Deep Tracking proposes to extend the paradigm of consensus-based collective cell response to the context of biology (see step 6 in Methods section). The underlying assumption is that the response of a given cancer cell to a given treatment can be quantified by aggregating, by a majority voting procedure, the classification results provided by all the pictures of the neighbouring cell tracks (cancer or immune cells according to the experiment). In other words, such a biologically-frames consensus strategy assumes that despite individual cell behaviour that can deviate from expected activity, the majority of cell motility characteristics can be used to demonstrate the unified hidden cancer cell message.

## Results

The high generalization capability of Deep Tracking approach is proved in this work by considering two different biological scenarios: breast cancer cell in immune-cancer crosstalk activities (CASE STUDY 1) and prostate cancer cell cultured with chemotherapeutic agents (CASE STUDY2). Generalization is proved by the use of different cell dimension, motility characteristics, and microfluidic environments (OOC vs Petri dishes). The validity of the classification problem is then performed using an experiment-independent testing procedure that can optimally demonstrate the actual generalizability of the approach. The selected testing approach ensures that trajectory pictures coming from the same video are not split between training and testing partition. More specifically, we used a two-fold testing procedure by dividing the videos of each case study in two partitions (in turn used for training and for testing and vice-versa). The same procedure is applied for both the case studies.

### Atlas preparation and classification task definition

**Figure 2:**
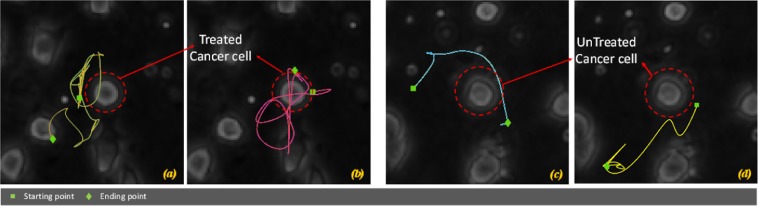
Four examples of trajectories of immune cells around a selected cancer cell: (**a**,**b**) treated cancer cells and (**c**,**d**) untreated cancer cells for CASE STUDY 1. Colours have been used only for the sake of track visualization.

*CASE STUDY 1:* we investigated the motility of immune cells in the proximity of cancer cells, without and with the drug trastuzumab, during an observation period of 72 hr; immune cells tracks are extracted and used to construct an atlas of images of immune cell tracks. The associated classification task was to evaluate the efficiency of drug treatment by analysing the “attractiveness” of tumor cells. More formally, the classification task is here to recognize experiments with treated cancer cells from experiments with untreated cancer cells by using as a “sentinel” the motility of the immune cells. The reference position here for the atlas construction is the centre of each tumor cell that is manually located. We verified that cancer cells were almost motionless and hence were located only in the first frame without the need of any further preprocessing step (i.e., segmentation, tracking, etc.). The underlying assumptions is that attractiveness of the cancer cell can be quantified by aggregating, by a majority voting procedure, the classification results provided by all the pictures of the immune cell tracks around the same cancer cell. In other words, such a biologically-frames consensus strategy assumes that despite individual immune cell behaviour that can deviate from expected activity, the majority of immune cell motility characteristics determines the general capacity of the tumor cell under the study to attract immune cells. Figure 2 shows some examples of selected cancer cells for (a)-(b) treated case and (c)-(d) untreated condition. Some related immune cells trajectories estimated by Cell Hunter software are also drawn. Colours are used only for the sake of visualization.

**Figure 3:**
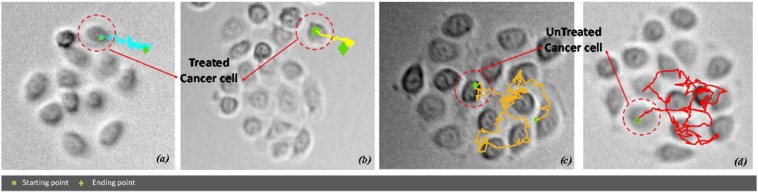
Four examples of trajectories of a cancer cell within a cluster: treated (**a,b**) and untreated conditions (**c**,**d**) for CASE STUDY 2. Colours have been used only for the sake of track visualization.

*CASE STUDY 2:* we investigated the motility of cancer cells, without and with the administration of the drug etoposide for 6hrs. The classification task was to recognize treated vs untreated cancer cells from their collective behaviour within the same cluster. The geometrical centre of each cluster (computed by combining the geometrical centres of all the tracks localized within) is used as reference point to construct the atlas. The motility of all the cancer cells belonging to the same cluster are jointly analysed. Again, the logic of consensus is used to support the final assessment of the whole cluster as treated or untreated, by aggregating the classification results provided by all the pictures of the cancer cell tracks within the same cluster. In other word, the crucial assumption embedded in the proposed model is the diverse role of cells according to the position in the clusters^23^. Cells belonging to the core seem to be less representative in motility variation effects than cells on the cluster rim, probably due also to confinement effects. This ability overcomes the less general assumption of cell-based drug effect isotropy (e.g., should all cells have been influenced by the same amount of drug in exactly the same manner?) that is directly connected to the assumed presence of diverse cell-roles (leader/follower) in collective migration^24^. Figure 3 shows some examples of selected cluster of cancer cells for (a)-(b) treated case with etoposide 50μM and (c)-(d) untreated condition. Some related immune cells trajectories estimated by Cell Hunter software are also drawn. Colours are used only for the sake of visualization.

### Accurate classification of cancer drug treatment effects

*CASE-STUDY 1*. In the first test, 12 different videos have been acquired as shown in Table 1a. In particular, VID1-VID6 belong to a first experiment while VID7-VID12 belong to a replicated experiment as highlighted by shaded green and yellow colours. Out of the videos, VID1, VID3, and VID5 (the same for VID7, VID9, and VID11) refer to the control case (without drug) while VID2, VID4, and VID6 (the same for VID8, VID10, and VID12) refer to the treated case (with drug). The number of the videos analysed increases the robustness and the generalizability of the method.

**Table 1:** Description of the two ATLAS compositions.

	EXPERIMENT 1						EXPERIMENT 2					
	**VID1**	**VID2**	**VID3**	**VID4**	**VID5**	**VID6**	VID7	VID8	VID9	VID10	VID11	VID12
	**CASE STUDY 1 (ATLAS 1)**											
Treatment	−	+	−	+	−	+	−	+	−	+	−	+
N. tumor Cells	10	10	10	10	10	10	10	10	10	10	10	10
N. tracks	200	200	200	200	200	200	200	200	200	200	200	200
**Total tracks**	**2400**											
	VID1	**VID2**	VID3	**VID4**	VID5	VID6	**VID7**					
	**CASE STUDY 2 (ATLAS2)**											
Treatment	+	−	−	+	+	+	+					
N. clusters	9	5	9	9	8	8	11					
N. tracks	39	45	154	31	40	30	60					
**Total tracks**	**399**											

VID stands for videos. Case study 1 atlas composition; Case study 2 atlas composition. Bolded videos indicate testing partition for each case study.

For each video, 10 cancer cells are manually identified for a total number of 120 cells (Table 1, Case study, 1 fifth row). In the neighbourhood of each cancer cell, immune cells are tracked and trajectories are used for the analysis (200 tracks around each cancer cell for a total number of tracks of 2400, Table 1, Case study 1, sixth row).

As mentioned before, two-fold testing has been applied in order to perform system assessment (yellow vs green shaded videos). Table 2 (Case study 1) lists the accuracy values (average and individual values obtained in each turn of the two-fold validation, calculated only on the testing partitions). Three approaches are compared, i.e., single cell analysis (no majority voting) (column 1), majority voting computed over the tracks around the same cancer cell (column 2), and majority voting applied over all the tracks in the same video (column 3).

**Table 2:** Classification accuracy values obtained for the two CASE STUDIES: CASE STUDY 1, CASE STUDY 2.

Accuracy Values	Single-cell analysis	Tumor-cell microenvironment analysis (majority voting)	Video-level analysis (majority voting)
Case Study 1			
**Deep Tracking**			
Training	**80% (78%–82%)**	**96% (95%–97%)**	**100%**
Test	68% (67%–69%)	91% (86%–96%)	100%
Case Study 2			
**Deep Tracking**			
**AccuracyValues**	**Single-cell analysis**	**Cluster-level analysis (majority voting)**	**Video-level analysis (majority voting)**
Training	**88% (87%**–**89%)**	**96% (94%**–**98%)**	**100%**
Test	82% (78%–85%)	92% (88%–95%)	100%

Diverse consensus levels have been considered. First column indicates single-cell analysis with no-consensus; second column indicates tumor-cell environment analysis where consensus by majority voting is performed over all the immune cells tracks in the neighbourhood of the same cancer cell (case study 1) or cancer cells tracks belonging to the same cluster (case study 2); third column indicates that majority voting has been performed at the video level by combining all the tracks within the same video. Balanced accuracy has been evaluated for all the situations to account for samples unbalance. The accuracy values within the brackets indicate the results obtained in each turn of the two-fold testing procedure. The average accuracy values are also reported.

Recognition accuracy is clearly improved after majority voting (second and third columns) indicating that the majority of trajectories around a given treated tumor cell has been recognized as significantly different from trajectories collected in the neighbourhood of untreated cancer cells. First of all, the result gives evidence of the fact there is an evident difference in immune cell motility approaching cancer cell in presence of drug, opening a new era for cell-motility based biosensors. Second, results obtained using a majority voting procedure demonstrate the fact that there is a dominant motility behaviour in immune cells around a cancer cells in presence of drug that can be exploited to evaluate increased cancer cell attractiveness in immunotherapy.

*CASE-STUDY 2*. In a second test, seven different videos were considered and divided into two partitions as indicated by the shaded green and yellow colours in Table 1b. In particular, VID2 and VID3 refer to the control case (without drug) whereas VID1 and VID4-VID7 refer to the treated case where a block replication drug was administered to the cancer cells with the aim to progressively reduce/stop cancer cell replication process. After plating in a Petri dish, cancer cells tend to cluster in a way that depends to drug administered. The number of clusters localized by Deep Tracking in each video are listed in Table 1 (Case study 2 total number of clusters equal to 59) along with the total number of tracks automatically extracted in each video (total number of tracks analysed equal to 399). Again, Deep Tracking is used to transform the atlas of the trajectories into numerical features and then an SVM classifier is trained using the two-fold testing strategy highlighted in colours in Table 1 (case study 2). Consensus aggregation is then applied to the labels assigned to the trajectories in the same cluster or to the trajectories within the same video.

Table 2 (case study 2) lists the accuracy values obtained in this case study. As above, average and individual accuracy values obtained in each turn are listed. As demonstrated in case study 1, majority voting is crucial in order to extract the dominant behaviour of the cells belonging to the cluster. Results indicated in columns 2 and 3 demonstrate that, more that individual cell motility, dominant migration characteristics become crucial in highlighting the cell behaviour towards the presence of drug. The high accuracy results obtained demonstrated the strength of the reasoning approach behind Deep Tracking methodology and the flexibility to be applied to diversified biological scenarios.

## Discussion

As a further demonstration of the effectiveness of the proposed approach with respect to the state of the art methodologies, we include direct comparison with alternative 1D convolutional deep learning methods (17), later *Method A*, as well as with classifiers using basic trajectory features, later *Method B* (see *Methods*). For the sake of comparison, we considered all the data included in the present manuscript, i.e., Case study 1 and 2. Table 3 summarizes the accuracy values obtained by the two comparative methods in the same three conditions as above in Table 2.

**Table 3:** Classification accuracy values obtained for the two CASE STUDIES (CASE STUDY 1, CASE STUDY 2) by applying METHOD A (time series analysis by 1D Deep Learning strategy as that described in^17^) and METHOD B (classifiers using basic trajectory features).

ACCURACY VALUES	Single-cell analysis	Tumor-cell microenvironment analysis (majority voting)	Video-level analysis (majority voting)
**CASE STUDY 1**			
METHOD A	59% (55–62%)	59% (55–62%)	59% (50–67%)
METHOD B	64% (60–70%)	79% (70–87%)	75% (67–83%)
**ACCURACY VALUES**	**Single-cell analysis**	**Cluster-level analysis (majority voting)**	**Video-level analysis (majority voting)**
**CASE STUDY 2**			
METHOD A	55% (54–56%)	55% (46–63%)	71% (67–75%)
METHOD B	65% (63– 66%)	70% (68–72%)	71% (67–75%)

The three diverse consensus levels of Tab. 2 have been considered. First column indicates single-cell classification results with no-consensus; second column indicates tumor-cell environment analysis where consensus by majority voting is performed over all the immune cells tracks in the neighbourhood of the same cancer cell (case study 1) or cancer cells tracks belonging to the same cluster (case study 2); third column indicates that majority voting has been performed at the video level by combining the prediction of all the tracks within the same video. Balanced accuracy has been evaluated for all the situations to account for samples unbalance. The accuracy values within the brackets indicate the results obtained in each turn of the two-fold validation procedure. The average accuracy values are also reported.

**Figure 4:**
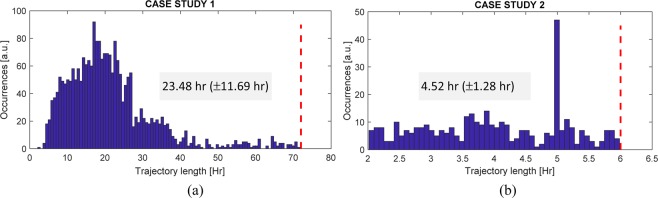
Distribution of track length for (**a**) Case Study 1 and (**b**) Case Study 2. The dashed red line indicates the observation time (**a**) 72 hr and (b) 6 hr.

As expected, the state of the art approaches reach very low performance with respect to the proposed method. This is due to the fact they use limited information about the input data such as 1D time series of coordinate or low-level trajectory statistics. More specifically, Regarding Method A, one serious limitation (originated by the 1D Deep Learning input fixed dimension) is related to the necessity to equalize the trajectory lengths of all the tracks involved. This is a problem already raised in^25^ for tracks comparison for which the solution of dummy points has been proposed. A dummy point is a point of the track artificially created and added to the track with the goal to extend the track length to a prefixed duration, typically the entire duration of the experiment. In our case, dummy points were added at the beginning and/or at the end of the track in order to have all the tracks starting at the first frame and ending at the last frame. After the revision of the commonly used approaches (e.g., zero-padding vs point repetition) we decided to replicate the initial and the last position of the track so to avoid abrupt changes of cell position during the video sequence). It is straightforward to note that the addition of such points affects the results obtained as demonstrated by the low accuracy values reported in Table 3. The effect is critical especially for Case study 1 when the observation time (72 hr) is much larger than the average duration of the trajectories (average 23 hr – std 17 hr, see Fig. 4).

On the other hand, for long biological experiments (up to some days), phenomena such as apoptosis (i.e., cell-death), mitosis (cell replication), or simply cell entering and exiting from the field of view, it is frequent to have heterogeneous track lengths. One of the strengths of Deep Tracking is that the 2D representation of a track allows us to overcome such an aspect and to treat all the tracks in the same way without altering their content.

In addition to this, the length heterogeneity demonstrates also the potential application of Deep Tracking to diverse biological scenario. Figure 4 shows the distribution of track length in the two Cases. The red lines set the observation time. It can be observed that especially in Case study 1, there is a large dispersion of track length in the range of 72 hr with an average length of 23 hr. This fact means that Deep Tracking is robust to length diversity, confuting the fact that the length of the trajectory can be a predictor itself.

The main limitation of Method B is the difficulty to determine which low-level features will be predictive of a certain phenotype and may allow the automatic discrimination of biological phenomena. This is due to the fact that specific kinematics features may be related to a very particular phenomena leading to an application-oriented scenario. For this reason, a method to determine relevant features at a high-level (i.e., those obtained by DL strategy) for a given classification task is therefore crucial.

The new approach proposed by Deep Tracking overtakes the limitations of very specific kind of analysis by providing a tool that can be applied to very diversified scenarios and a DL model able to directly use the visual representation of what is under the microscope in order to reveal the hidden messages that cell trajectories carry on during the experiment.

To conclude, first of all, we proved that the appearance of cell trajectories itself conveys the information of the underlying biological phenomenon (drug administration, block replication effects, and so on), instead of detailed features extracted from the motility and the morphology of cells during the video sequences, as widely discussed before.

Secondly, the collaborative task represented by the combined activities of immune cells in the neighbourhood of cancer cells or of multitude of cancer cells within the same cluster can be effectively evaluated by computing the majority of the responses of all the agents within the collectivity. To this regard, we demonstrated that the majority of the responses is more reliable than simply averaging the responses of a synergic group of cells.

## Methods

The software that we have developed for this kind of analysis and the detailed results are described in the following sections.

*Step1. Video acquisition*. Time-lapse microscopy generates a video sequence of the motile cells in the context of the experimental environment. Video sequence is composed by a series of sequential images each related to a static picture of the environment. Time-resolution (or frame rate) refers to the time-lag between two consecutive frames and pixel resolution refers to the equivalent dimension of an image pixel that is therefore the smallest amount of information distinguishable in the digital image. Typical time-resolution in cell-microscopy is within the [1,4] *min* range and pixel resolution is within [0.5-2]μm. Such parameters are crucial for reliable cell motility understanding. In the considered experiments, we have considered time-resolution of 1 and 2 min^12,26^ and pixel resolution of 0.66 and 0.64 μm^12,26^, for case study 1 and 2, respectively (see Results section).

*Step2. Cell localization*. Cells have to be localized within each frame and, in case of multi-population (e.g., the case of immune and cancer cells in the same microenvironment), different cell types have to be distinguished in an automatic way. Cells can be located using free available segmentation tools (e.g., ImageJ software^22^) or dedicated proprietary software. In our experiments, we developed a specific software for the task called *Cell Hunter*, whose mathematical details can be found in^12,27^. Cell Hunter includes the segmentation of circular-shaped objects using Circular Hough Transform^28^ with radius tolerance tuned according to a pre-estimation of cell radius for different populations, e.g. cancer cell radius about three times peripheral blood mononuclear cell (PBMC) radius. The position of each cell is hence coded into coordinates (*x,y*) and radius *r* for each time frame.

*Step3. Cell tracking*. Cells localized in a frame have to be linked to cells located in the next frame. The need to simultaneously link all the cells located forced us to solve an Optimal Sub-pattern Assignment Problem using the Munkres algorithm^29^ yielding the globally best possible pairing among located objects based on a given assignment cost. According to previous studies^30^, it is reasonable to assume cells motion to be a random walk with time-varying drift and volatility. Let us indicate with $(x(t),y(t))$ the coordinate of a given cell at time *t*, then we may indicate the displacement in each direction at time *t* + 1 as

1 $$\{\begin{array}{c}\triangle x(t)=x(t+1)-x(t)={\mu }_{x}(t)+{\sigma }_{x}(t)\bullet w(t)\\ \triangle y(t)=y(t+1)-y(t)={\mu }_{y}(t)+{\sigma }_{y}(t)\bullet w(t)\end{array}$$

where $w(t)$ is a White Random Process, ${\mu }_{x}(t)$ and ${\mu }_{y}(t)$ are two independent time-varying drift terms, while ${\sigma }_{x}(t)$ and ${\sigma }_{y}(t)$ are two independent time varying volatility terms. Drift terms indicate the displacement in the 2D domain of the cell from a position to the next one, while the volatility is related to the dispersion of the displacement of the cell at each movement. It is straightforward to demonstrate that drift and square volatility parameters are the expected value and the expected variance of random displacement $\triangle x(t)$ and $\triangle y(t)$ respectively. Assuming local ergodicity of processes $\triangle x(t)$ and $\triangle y(t)$ in a limited time interval T, we then provide estimates for ${\mu }_{x}(t)$, ${\mu }_{y}(t)$, ${\sigma }_{x}^{2}(t)$, and ${\sigma }_{y}^{2}(t)$ as follows:

$${\hat{\mu }}_{x}(t)=\frac{1}{T}\mathop{\sum }\limits_{s=t-T}^{t}\triangle x(s){\hat{\mu }}_{y}(t)=\frac{1}{T}\mathop{\sum }\limits_{s=t-T}^{t}\triangle y(s)$$

2 $${\hat{\sigma }}_{x}^{2}(t)=\frac{1}{T}\mathop{\sum }\limits_{s=t-T}^{t}{(\triangle x(s)-{\hat{\mu }}_{x}(s))}^{2}{\hat{\sigma }}_{y}^{2}(t)=\frac{1}{T}\mathop{\sum }\limits_{s=t-T}^{t}{(\triangle y(s)-{\hat{\mu }}_{y}(s))}^{2}$$

Moreover, assuming independence between displacements $\triangle x(t)$ and $\triangle y(t)$, the joint probability density function of the two random processes $\triangle x(t)$ and $\triangle y(t)$ is given by

3 $${f}_{{xy}}(\triangle x(t),\triangle y(t))=\frac{1}{2\pi {\hat{\sigma }}_{x}(t){\hat{\sigma }}_{y}(t)}{e}^{-\left(\frac{{(\triangle x(t)-{\hat{\mu }}_{x}(t))}^{2}}{2{\hat{\sigma }}_{x}^{2}(t)}+\frac{{(\triangle y(t)-{\hat{\mu }}_{y}(t))}^{2}}{2{\hat{\sigma }}_{y}^{2}(t)}\right)}$$

Therefore, the cost matrix for solving the assignment problem includes at position $(n,m)$ the inverse of the probability (given by Eq. 3) that cell numbered as $n$ in frame $i$ has moved to the position occupied by cell numbered as $m$ in frame $i+1$. Hence, the minimum cost is equivalent to the maximum overall probability of assignment. The tracking step produces a given number of tracks for each cell population.

*Step4. Construction of the atlas of trajectories*. Each track is coded as $(t,x(t),y(t))$^31^. Provided a reference point $({x}_{c},{y}_{c})$ then we construct a 2D visual representation of the track by marking to 1 each pixel located at $({x(t)-x}_{c},{y(t)-y}_{c})$ within a given region size around the reference point. In this way, each track position is referred to a given location of interest that depends on the biological experiment under study (e.g., geometrical center of the track itself, geometrical center of all the tracks within a cluster, geometrical centre of tumor cell track for interaction studies, etc.).

The black and white representation of the single track (also named binary image or BW image in the image processing lexicon) produces a 2D projection of the track along time. An image with a singleton white marker denotes a stopped cell, as well as a linear pattern on the image denotes a drift motility, etc. Computer simulations allows to repeat the procedure for thousands of cells in a few seconds, thus enormously increasing the human capability of recording cells simultaneously moving in the environment. After applying the visual coding to all the tracks of the experiments of interest, a so-called *visual atlas of trajectories* is constructed.

**Figure 5:**
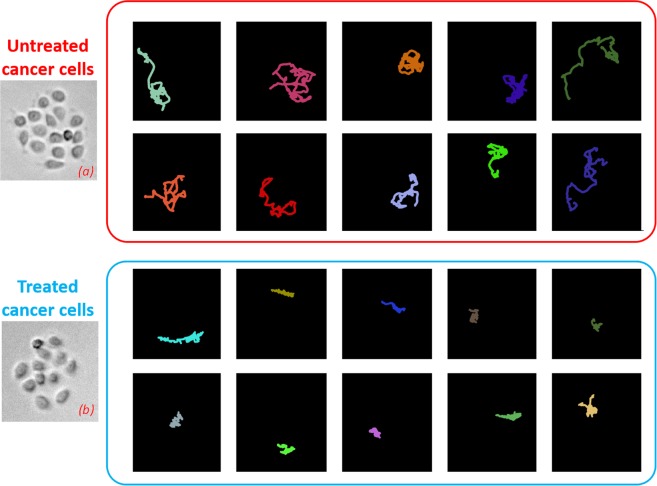
Examples of atlases of trajectory pictures for CASE STUDY 2. (**a**) Cluster of untreated cancer cells. (**b**) Cluster of treated cancer cells with etoposide agent 50μM. Colours are used only for the sake of visualization, since pictures of trajectories are represented by black & white images.

Figure 5 shows some examples of atlases for two different cluster of cells in CASE STUDY 2. Cluster in (a) belongs to an untreated case and cluster in (b) relates to a treated case with 50μM of etoposide. Colours are used only for the sake of visualization, since pictures of trajectories are represented by black & white images.

*Step5. Feature extraction (by CNN network)*. On the inspiration of the image style transfer^25^, we consider here a pre-trained DL architecture based on Convolutional Neural Network (CNN), that was used in competition ImageNet Large Scale Visual Recognition Challenge^14^. AlexNET has been trained on 15 M labelled images on more than 22 K categories. AlexNET allows to extract the style of the atlas by mapping the images into a set of descriptors that best represent the general core of the atlas (this procedure is also referred as *Transfer Learning)*. Such descriptors may be used for different classification tasks in order to discriminate the biological behaviours of interest for the cells under observation. To assure more versatility to the method, we avoided a further fine retuning of the AlexNET architecture that would lead to the risk of overlearning phenomena. In the present work, we selected the *pool2* layer (layer 9 in the AlexNET architecture) in which resulting features for each image are 43264. Most of the features are then reduced by unsupervised feature selection procedure based on the variance criterion (i.e., small feature variability implies rejection). The choice of a specific layer is motivated by the fact that the lower the information content of the image the higher the level for representation, to avoid excessive loss of information due to pooling and dropout procedures.

*Step6. Classification task*. Machine learning allows biologists to construct models of cell taxonomy according to the analysis performed in specific experimental conditions^13,32^. Drug efficacy, block of replication effects, apoptosis and/or mitosis rate, cell subtyping, are only a few examples deriving from cell motility studies. In the proposed case studies, features from the inner pooling layer (i.e., pool2) of the pre-trained AlexNET DL-CNN were extracted for the classification task. The kind of the atlas we extract do not motivate to go further in inner layers. We realized that pool2 was optimal to recover local patterns (such as change of direction or linear motion) but at the advantage of a reduction in atlas dimension. Then, Support Vector Machine (SVM) with linear kernel have been implemented. SVM represents a very consolidated machine learning technique, suitable to prevent overfitting problem thanks to the logic of support vectors^33^. For the aim of model training and testing, treated experiment were labelled as positive (drug administration in OOC microenvironment or in a Petri Dish) while untreated experiments were marked as negative (no drug). For each of the two atlases, we have validated the classification model by a two-fold testing strategy. The dataset of each case study has been divided into two independent experiments (case study 1) or independent set of experiments (case study 2). In turns, one of the two partitions is used to construct the model while the remaining one is used for testing. The two partitions are then exchanged. The average number of correctly classified cancer cells in the two classes (i.e., treated and untreated) in each case study is then computed as accuracy value and reported herein.

*Step 7. Consensus aggregation*. In a “wisdom of crowd” logic^34^, consensus based technique can then be used to aggregate results extracted from labelling (treated vs untreated) assigned by the classification model to immune cell trajectories around the same tumor cell (Case study 1) or to cancer cells in the same cluster (Case study 2), respectively. The main rationale behind this way to proceed is related to the awareness that individual cell motility alone cannot be representative of a given phenomenon, whose dynamics can be instead more realistically supported by the majority of cell responses. To apply this logic, for each cancer cell in case study 1 we computed the majority of the labels assigned to the immune cell trajectories in its neighborhood or for a more robust approach the majority of labels assigned to all the immune cell tracks in the entire video. For case study 2, the majority is computed over the labels assigned to the cancer cells in the same cluster to have the dominant behavior of the entire cluster of cells. Again, for a more robust approach, the majority of the labels assigned to all the cancer cell trajectories within the same video is also computed.

### Method for comparative benchmark

To validate the potentialities of the proposed method, we have compared its performances with those obtained with two golden standard methods, called briefly Method A and Method B, for this kind of problem. For the sake of comparison, the same two-fold testing framework is used for both the methods.

Method A combines a Recurrent Neural Network (RNN) with 1D Convolutional Neural Network (CNN) feature extractors in a unique architecture, where ‘1D’ refers to apply CNNs to one-channel time series. If, on the one hand, CNN features extractors provide an unsupervised feature learning, on the other hand, RNN units, such as Long Short-Term Memory (LSTM) units, may capture the temporal dependencies within the input time series. Specifically, in the case under examination, cell trajectories in input may be considered as one-channel time series, disposed in a (time, *x*/*y* coordinate) matrix of fixed dimensions, with t as spatial dimension and x/y coordinate as one channel dimension.

Briefly, the CNN-RNN classification architecture is composed by four convolutional layers with one spatial dimension and one channel dimension, followed by an LSTM layer with n = 256 hidden units and two fully-connected layers of 256 and 128 units, respectively, paired with a rectified linear unit activation. The architecture lasts with a fully-connected layer with number of units equal to the number of classes paired with a softmax activation. Refers to^17^ for more details.

Method B extracts the 28 standard kinematics descriptors proposed in^26^ for each trajectory and use them within an SVM classifier with linear kernel^33^ and default setting. The 28 descriptors are obtained by computing statistical moments over the following eight low-level features: tangential speed magnitude, *f*_1_, track curvature, *f*_2_, angular speed magnitude, *f*_3_, turning angle, *f*_4_, distance to track centre, *f*_5_, average distance from the cancer/cluster centre, *f*_6_, diffusion coefficient, *f*_7_, and directional persistence, *f*_8_. In particular, to account for the variability of features *f*_1_- *f*_5_ along the track, we derived first, second, third and fourth statistical moments in addition to the Shannon entropy computed along time. Such high-level computation leads to a total of 25 high-level descriptors in addition to the remaining three low-level descriptors, *f*_6_–*f*_8_. Further mathematical details can be found in^26^. The main strength of such approach is that it is length-independent and hence allows to analyse trajectories with heterogeneous duration. On the other hand, a unique feature is often unrepresentative of very complex cell behaviour especially in very long observation period.

## Supplementary information

Supplementary Information.

## Data Availability

All the software codes and a detailed user manual with an example of application can be found at https://cloudstore.bee.uniroma2.it/index.php/s/wybpcKRkMSbL4qN, password: utov2019.
