# Supplementary material for: Discovering the hidden messages within cell trajectories using a deep learning approach for in vitro evaluation of cancer drug treatments
Source: Sci Rep. 2020 May 6;10:7653. doi: 10.1038/s41598-020-64246-3 (PMC7203117; doi:10.1038/s41598-020-64246-3)
Supplement: Supplementary file 1 — Supplementary Information. [file 41598_2020_64246_MOESM1_ESM.docx]

**SUPPLEMENTARY INFORMATION**

**Discovering the hidden messages within cell trajectories using a deep learning approach for in vitro evaluation of cancer drug treatments**

**A. Mencattini^1^, D. Di Giuseppe^1^, M.C. Comes^1^, P. Casti^1^, F. Corsi^2^, F.R. Bertani^3^, L. Ghibelli^4^, L.Businaro^3^, C. Di Natale^1^, M.C. Parrini^5^, E. Martinelli^1*^**

^1^Department of Electronic Engineering, University of Rome Tor Vergata, Rome, Italy

^2^Department of Chemical Science and Technologies, University of Rome Tor Vergata, Rome, Italy

^3^Institute for Photonics and Nanotechnology, Italian National Research Council, 00156 Rome, Italy

^4^Department of Biology, University of Rome Tor Vergata, Rome, Italy

^5^Institute Curie, Centre de Recherche, Paris Sciences et Lettres Research University, 75005 Paris, France

*martinelli@ing.uniroma2.it

**EXPERIMENTAL DETAILS**

To demonstrate the feasibility and versatility of Deep Tracking functionalities, we will present two different case studies:

**CASE STUDY 1: Organ-On-Chip (OOC)**

We consider an Organ-On-Chip (OOC) framework which recapitulates the 3D microenvironment of HER2+ breast cancer tumor ecosystem [1] (cancer cells, fibroblasts, endothelial and immune cells) embedded in extra-cellular matrix in absence or presence of anticancer drug trastuzumab, a targeted antibody therapy directed against HER2 receptor. The cell death rate is roughly 2.5% per day for untreated cancer cells and 30% per day for trastuzumab-treated cells, in presence of immune cells. It is important to remark that it did not affect the capacity of the method to correctly classify cells according to presence or not of the drugs.

More details about the experiments can be found in [1]. As an example, see Video1 at <https://cloudstore.bee.uniroma2.it/index.php/s/wybpcKRkMSbL4qN>, password: utov2019.

**CASE STUDY 2:** **cancer cell motility**

Standard 2D cultures in Petri dishes were performed to investigate the secondary effect of etoposide (standard chemotherapeutic agent, topoisomerase II inhibitor) as motion inhibitor of prostate cancer cells PC-3 [2,3]. PC-3 prostate cancer cells were grown at 37°C in RPMI 1640 medium supplemented with 10% fetal bovine serum FBS (Gibco), 2 mg/mL L-glutamine, 100 IU/mL penicillin and streptomycin (Euroclone) in a humidified atmosphere of 5% CO_2_ in air. Cells were seeded in 35 mm petri dishes (Greiner) and grown to 40-60% confluence. Cells were treated with the etoposide (Sigma) at the final concentrations of 0.5μM, 5μM or 50μM and immediately analysed by time lapse microscopy for 6 hrs. In this work, we did not discriminate among different drug concentrations, but we relied to the binary problem associated (drug vs no-drug). As an example, see Video2 at <https://cloudstore.bee.uniroma2.it/index.php/s/wybpcKRkMSbL4qN>, password: utov2019.

**REFERENCES**

1. Nguyen, M., De Ninno, A., Mencattini, A., Mermet-Meillon, F., Fornabaio, G., Evans, S. S.,et al., (2018). Dissecting Effects of Anti-cancer Drugs and Cancer-Associated Fibroblasts by On-Chip Reconstitution of Immunocompetent Tumor Microenvironments. *Cell reports*, 25(13), 3884-3893.
2. De Nicola, M., Bruni, E., Traversa, E., & Ghibelli, L. (2017). Slow release of etoposide from dextran conjugation shifts etoposide activity from cytotoxicity to differentiation: a promising tool for dosage control in anticancer metronomic therapy. Nanomedicine: Nanotechnology, *Biology and Medicine*, 13(6), 2005-2014.
3. Di Giuseppe, D., Corsi, F., Mencattini, A., Comes, M. C., Casti, P., Di Natale, C., et al., (2019). Learning cancer-related drug efficacy exploiting consensus in coordinated motility within cell clusters. IEEE Transactions on Biomedical Engineering. 1-3, doi: 10.1109/TBME.2019.2897825.
